# New Insights into HIV Life Cycle, Th1/Th2 Shift during HIV Infection and Preferential Virus Infection of Th2 Cells: Implications of Early HIV Treatment Initiation and Care

**DOI:** 10.3390/life14010104

**Published:** 2024-01-09

**Authors:** Joseph Hokello, Kratika Tyagi, Richard Oriko Owor, Adhikarimayum Lakhikumar Sharma, Alok Bhushan, Rene Daniel, Mudit Tyagi

**Affiliations:** 1 Department of Biology, Faculty of Science and Education, Busitema University, Tororo P.O. Box 236, Uganda; hokello.joseph@kiu.ac.ug; 2 Department of Biotechnology, Banasthali Vidyapith, Jaipur 304022, India; lsmst21030_kratika@banasthali.in; 3 Department of Chemistry, Faculty of Science and Education, Busitema University, Tororo P.O. Box 236, Uganda; richoriko@gmail.com; 4 Center for Translational Medicine, Thomas Jefferson University, 1020 Locust Street, Philadelphia, PA 19107, USA; lakhikumarsharma.adhikarimayum@jefferson.edu (A.L.S.); rene.daniel@jefferson.edu (R.D.); 5 Department of Pharmaceutical Sciences, Jefferson College of Pharmacy, Thomas Jefferson University, Philadelphia, PA 19107, USA; alok.bhushan@jefferson.edu

**Keywords:** HIV, HIV life cycle, viral transmission, T-helper cells, cytokines

## Abstract

The theory of immune regulation involves a homeostatic balance between T-helper 1 (Th1) and T-helper 2 (Th2) responses. The Th1 and Th2 theories were introduced in 1986 as a result of studies in mice, whereby T-helper cell subsets were found to direct different immune response pathways. Subsequently, this hypothesis was extended to human immunity, with Th1 cells mediating cellular immunity to fight intracellular pathogens, while Th2 cells mediated humoral immunity to fight extracellular pathogens. Several disease conditions were later found to tilt the balance between Th1 and Th2 immune response pathways, including HIV infection, but the exact mechanism for the shift from Th1 to Th2 cells was poorly understood. This review provides new insights into the molecular biology of HIV, wherein the HIV life cycle is discussed in detail. Insights into the possible mechanism for the Th1 to Th2 shift during HIV infection and the preferential infection of Th2 cells during the late symptomatic stage of HIV disease are also discussed.

## 1. Introduction

HIV is classified into HIV type one (HIV-1) and type two (HIV-2) [1,2,3]. In overall genomic nucleotide sequence homology, HIV-2 is approximately 60% homologous to HIV-1 but less virulent than HIV-1. The mechanistic differences in virulence and pathogenesis are unknown. Whereas HIV-1 is predominantly found throughout the world, HIV-2 is primarily confined to West Africa due to lower infectivity and transmission rates [1]. However, if untreated, HIV-2 can also progress to acquired immunodeficiency syndrome (AIDS) and eventual death. Nevertheless, HIV-2 is beginning to spread to other regions of the World, such as India, Europe and the United States. The observed difference in global distribution between HIV-1 and HIV-2 is due to the high infectivity of HIV-1, which makes it more transmissible than HIV-2 [4]. Several cross-species transmission events occurred from nonhuman primates to humans, giving rise to multiple HIV-1 groups and HIV-2. For instance, HIV-1 group M, with the most diverse viral subtypes, has been traced to the simian immunodeficiency virus, SIVcpz, which infects chimpanzees (*Pan troglodytes*). Other SIVs from chimpanzees have been independently transmitted to humans, giving rise to HIV-1 group N and O, while group P is believed to have been transmitted from gorillas [5]. On the other hand, molecular analysis revealed that HIV-2 and SIVsm from sooty mangabeys (*Cercocebus atys*) are closely related to each other and to SIVs from macaques. The close phylogenetic relationship and sequence similarity indicate that, indeed, HIV-2 is a result of zoonotic transmission from SIVsm to humans in the West Africa [6]. Even though HIV-1 and HIV-2 are distinct viruses (Table 1), they are also similar in a number of ways, including similarities in their gene arrangement, cellular replication cycles and clinical outcomes leading to AIDS. However, one of the major clinical differences between HIV-1 and HIV-2 is that infection with HIV-1 progresses much faster to immunodeficiency than infection with HIV-2, which occurs much more slowly. Consequently, long-term non-progressor phenotypes are more commonly observed in HIV-2 infection. HIV-1 is more infective and highly transmissible than HIV-2, and it is more pathogenic and establishes higher virus loads during the asymptomatic phase of infection, referred to as clinical latency, resulting in faster disease progression [7]. HIV-1 encodes a *vpu* gene (unlike HIV-2, which encodes the *vpx* gene) that is vital during HIV-1 virion release from the infected cell surface during egress. *vpu* promotes the trafficking of Env proteins to the cell surface for virion assembly through the degradation of the CD4 molecules within the CD4-Env protein complex, which gets trapped in the endoplasmic reticulum (ER) during viral protein translation and synthesis. However, the function of the HIV-2 vpx is to counter the inhibitory effects of the sterile alpha motif and HD domain 1 (SAMHD1), which is a cellular restriction factor. SAMHD1 restricts HIV-2 replication through inhibition of the reverse transcription process [8,9].

## 2. Genomics and Proteomics of HIV-1

**Figure 1:**
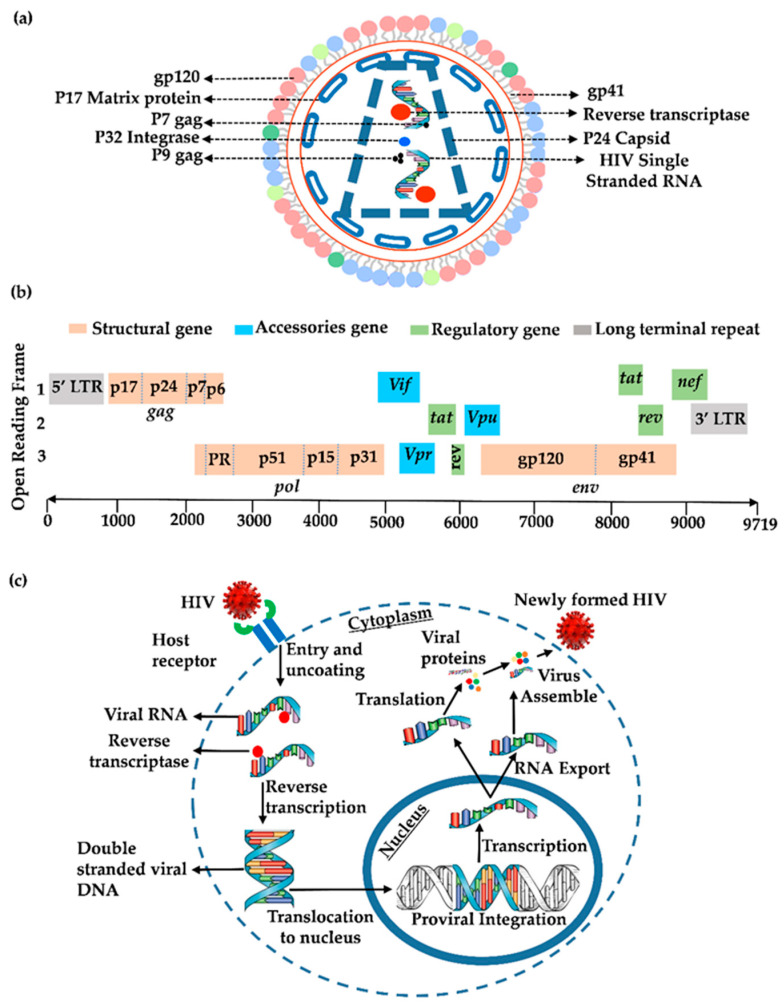
(**a**) Schematic representation of the HIV-1 virion. HIV-1 entry into a target cell is mediated through the surface glycoprotein 120 (gp120) and the transmembrane gp41 embedded within the viral envelope. The inner side of the envelope surrounds the capsid. Enclosed within the viral capsid are viral genomic RNA (gRNA), reverse transcriptase, protease and integrase enzymes, which are required for the successful integration of the provirus into the host chromosome. (**b**) The HIV-1 genomic Structural organization. The HIV-1 consists of nine Open Reading Frames (ORFs) flanked via Long Terminal Repeats (LTRs) on both ends. HIV-1 proviral gene expression is initiated from the LTR, which harbors multiple cis-regulatory elements for different transcription factors that regulate the expression of the HIV-1 genes. (**c**) The HIV-1 Life cycle. HIV-1 virion entry into a target cell occurs through the interaction of gp120 with CD4 receptor and gp41 with co-receptors. Upon entry into the cell cytoplasm, the nucleocapsid core uncoats, releasing the viral gRNA, which is reversed-transcribed into cDNA and translocated to the nucleus for integration into the genomic DNA of the host cell. Once integrated, the provirus is expressed, during which the viral mRNAs are exported and translated into viral proteins within the cell cytoplasm. The gRNA, along with viral proteins, is processed. Nef suppresses HIV-1 super-infection by reducing the HIV-1 receptor and co-receptors at the cellular surface. Furthermore, the Major Histocompatibility Complex type I (MHC-I), mainly HLA-A and HLA-B, are down-regulated by Nef, thereby facilitating immune evasion and protecting the virus from cell-mediated immune responses.

HIV, the causative agent of AIDS, is an enveloped, icosahedral RNA virus that belongs to the lentivirinae subfamily and the lentiviridae family of retroviruses [23,24,25]. HIV has two copies of the RNA genome, which is positive-sense, single-stranded, with approximately ~9700 nucleotides encased within an enveloped virion measuring ~120 nm in diameter (Figure 1a). The genome of HIV-1 is made up of nine open reading frames (ORFs), of which three code for Gag, Pol and Env polyproteins, which are proteolytically cleaved by furin protease, a cellular enzyme, to generate functional individual HIV-1 proteins (Table 2). Protein products resulting from the cleavage of the Gag polyprotein include the matrix (MA), capsid (CA), p7 and p6, all of which are structural proteins forming the HIV-1 virion core [26]. Structural proteins are required for the assembly of virus-like particles during virus replication. The roles played by the Gag protein during the viral life cycle include viral assembly, maturation and early post-entry steps in virus replication [27]. In 2004, the Sodroski group demonstrated that a cellular protein called tripartite motif 5 alpha (TRIM5α) restricts HIV-1 replication through proteosome-independent degradation of the cytoplasmic HIV CA [28]. However, it was later demonstrated that, unlike the nonhuman primate TRIM5α, human TRIM5α exhibited weak antiviral activity. The HIV-1 *env* gene codes for the viral protein Env, a 160 kD glycoprotein (gp160), necessary for entry into target host cells. Following translation, the Env polyproteins are proteolytically cleaved into viral envelope glycoprotein 120 (gp120) and the corresponding transmembrane glycoprotein 41 (gp41) [29]. The gp120 subunit from the N-terminus is completely outside the viral envelope, while the C-terminal gp41 subunit is retained inside the viral envelope. The gp41 has a membrane-spanning domain and an extracellular domain that mediates the conformational change necessary for fusion.

Three viral enzymes, namely reverse transcriptase (RT), protease (PR) and integrase (IN), which are encapsulated within the HIV virion particle, arise from the Pol polyprotein and are required for virus replication [26]. In addition to the Gag, Pol, and Env coding sequences, HIV-1 uniquely codes for an additional six ORFs (Figure 1b). Three virus regulatory proteins (Nef, Rev and Tat) are required for virus replication, while another three accessory proteins (Vpr, Vpu and Vif) are critical to HIV-1 as virulence factors in vivo, though the accessory proteins are dispensable for virus growth in many ex vivo systems [26].

A 27 KDa Nef protein is associated with the cellular membrane through myristylation at its N-terminus. Previously, Nef was believed to negatively regulate HIV-1 replication, which led to the coining of the name “negative factor”. However, several reports later demonstrated that Nef functions to enhance virus replication through modulation of cell receptor signaling [42,43]. Nef also induces rapid endocytosis and lysosomal degradation of CD4 molecules on HIV-1 infected cells, which are the main HIV-1 receptors for viral entry. Moreover, HIV-1 co-receptors, including CCR5 and CXCR4, are also down-regulated by the Nef protein. The host protein, SERINC, is an HIV restriction factor that inhibits virus infectivity when incorporated into the viral envelope, and the inhibitory effects of the SERINC protein occur through interaction with HIV-1 Nef. Recently, it was demonstrated that the SERINC protein is a membrane transporter that causes membrane asymmetry through flipping lipids, which strongly correlates with changes in envelope conformation and loss of viral infectivity [44].

Rev, a 19 KDa protein, is encoded by the two-exon viral mRNA transcripts. The first set of mRNAs produced during HIV-1 transcription is multiply spliced and encodes regulatory proteins Nef, Tat and Rev. Rev facilitates the nuclear export of unspliced viral RNAs. To perform its function, Rev requires Rev Responsive Elements (RRE), an RNA sequence that allows Rev binding to the mRNA. Given the fact that RRE is spliced out from multiply spliced HIV-1 mRNAs, Rev does not export multiply spliced HIV mRNAs out of the cell nucleus where transcription occurs [26,45]. Therefore, Rev performs a crucial role in exporting unspliced HIV RNA transcripts out of the nucleus. Within the cell cytoplasm, the complete HIV-1 gRNA then assembles with other viral proteins to form new HIV progenies, which then bud out of the cell membrane and undergo maturation to maintain virion infectivity.

The long terminal repeat (LTR) of HIV-1 harbors several *cis*-regulatory elements that are critical for RNA polymerase II (RNAP II)-mediated transcription, including binding sites for the nuclear factor kappaB (NF-κB) [46,47] and the nuclear factor of activated T-cells (NFAT) [48]. Despite the critical importance of these factors in activating transcription from the HIV LTR, HIV transcription is less efficient, and transcribing RNAP II is less processive without the HIV-1 Transactivator of transcription (Tat) protein [49,50,51,52]. When Tat is absent, RNAP II can only transcribe less than 100 nucleotides downstream from the transcription start site, and most transcripts terminate before completion. However, when viral Tat protein is present, it enhances both the quality and quantity of HIV-1 transcription. Tat selectively binds the transactivation response RNA (TAR) sequences, a stem-loop bulge structure. The TAR sequences are present at the 5′ terminus of each HIV-1 transcript. When Tat binds to the TAR sequence of the new HIV-1 mRNA transcripts, it promotes the recruitment of the positive transcription elongation factor b (P-TEFb) complex. Subsequently, the subunit of P-TEFb called cyclin-dependent kinase 9 (CDK9) then catalyzes the phosphorylation of the C-terminal domain (CTD) of RNAP II, thereby making it more processive (elongation proficient). Consequently, Tat augments HIV-1 transcription more than 100-fold, primarily by enhancing the HIV-1 transcriptional elongation phase [26,52,53,54,55,56].

Vif, a 23 KDa protein, is required for the production of mature infectious virions. Virions that lack Vif exhibit defective infectivity when prepared in nonpermissive or semipermissive cell types. Unlike permissive cells, non and semipermissive cells express host cellular restriction factors called human apolipoprotein-B mRNA-editing enzyme polypeptide-like 3 (APOBEC3) proteins, which inhibit the replication of HIV-1 virions lacking the Vif protein [57]. When Vif-deficient HIV-1 replicates, APOBEC3 protein is incorporated into new virions, which triggers massive conversion of deoxycytidine (dC) to deoxyuridine (dU) in the noncoding strand of viral cDNA during reverse transcription [57]. Deamination of the noncoding strand of viral cDNA consequently inactivates the synthesis of the coding strand of cDNA during reverse transcription and thus impairs the reverse transcription of HIV. However, the Vif protein rescues HIV reverse transcription by mediating the proteolysis of cellular APOBEC3 proteins through proteasomal degradation and enables HIV-1 to replicate in non and semipermissive cell types. The post-translational modification also regulates the activity of HIV-1 Vif; for instance, a mutation in serine phosphorylation sites (Ser144) causes a defective viral infectivity [58].

Vpr, a 14 KDa basic protein, is associated with HIV-1 virions via interactions with the C-terminus of the Gag protein. Vpr mediates the nuclear import of the pre-integration complex (PIC) of HIV-1 by tethering the PIC to nuclear-import proteins and modulating the cell cycle [45]. Consequently, the Vpr protein enables active nuclear transport of HIV PIC without cell division. Unlike other retroviruses, lentiviruses, including HIV, do not rely on nuclear envelope breakdown during mitosis to enter the nucleus. Thus, Vpr uniquely allows HIV-1 to infect a wide array of cell types, including cells in the nondividing phase. Furthermore, Vpr induces G2 cell-cycle arrest and subsequently kills T cells through programmed cell death or apoptosis [59]. Moreover, Vpr is reported to influence mutation rates during the synthesis of viral DNA [60].

Vpu, a 16 KDa membrane phosphoprotein, is unique to HIV-1. Vpu is not found in HIV-2 or Simian Immunodeficiency virus (SIV) except for SIVcpz, which is the closest relative of HIV-1 [26]. The Bieniasz group initially identified Tetherin, a cellular protein that inhibits the release of HIV-1 virions and other enveloped viruses from the cell surface during egress. Tetherin acts by retaining the virions of HIV-1 on the cell surface [61]. However, during HIV-1 virion release from the surface of infected cells, Vpu counteracts the effects of Tetherin during budding and egress. Vpu is also known to promote the trafficking of envelope proteins to the surface of cells for virion assembly through degradation of the CD4 molecules within the CD4–Env protein complex, which gets trapped in the endoplasmic reticulum (ER) during viral protein translation and synthesis. Post-translational modification abrogates the Vpu effects on CD4 degradation. Casein kinase-2-related protein phosphorylates Vpu on Ser52 and Ser56, such that mutation at these positions decreases the levels of CD4 degradation [62].

## 3. HIV Lifecycle

### 3.1. Entry and Reverse Transcription

HIV specifically infects cells that express CD4 molecules on their surface, including macrophages, helper T cells and microglial cells. During entry, HIV interacts with both the CD4 receptor [63] and co-receptor, either CCR5 [64] or CXCR4, through its envelope glycoproteins (gp120) [65]. On the virus surface, the HIV gp120 forms trimers, with each monomer consisting of the gp120 and gp41 subunits. The gp120 domain interacts with the CD4 molecule and undergoes conformational changes on target cells that allow gp120 to subsequently interact with viral CCR5 or CXCR4 co-receptors [66]. Trimolecular complex formation stabilizes the binding of the virus and triggers additional changes in conformation in the gp41, which promotes fusion with the membrane and entry into the cell cytoplasm [66]. Following fusion events, the capsid core of HIV-1 gets degraded in a process referred to as uncoating, which was previously thought to occur within the cytoplasm to release a high molecular weight reverse transcription complex (RTC) [67]. However, recent reports have revealed that uncoating occurs in the nucleus of the target cell [68,69]. Apart from the CD4 molecule and the chemokine co-receptors, other HIV receptors have been reported. For instance, Dendritic cell (DC)-specific intracellular adhesion molecule (ICAM)-3-grabbing non-integrin (DC-SIGN), which is a c-type lectin, is important in disseminating HIV-1 to T cells by DCs, especially during HIV-1 mucosal transmission [70,71]. Similarly, Langerhans cells (LCs), particularly immature LCs, which are the first DC subsets that encounter HIV in the mucosa, are reported to inhibit the transfer of HIV-1 to T cells through interaction with a receptor called langerin, a c-type lectin [72,73,74]. Cicala et al. also reported that high levels of integrin α4β7, which is a gut-homing receptor, are expressed by mucosal CD4+ T cells. They observed that in the mucosa, the HIV-1 envelope first interacts with α4β7 on CD4+ T cells in close association with CD4 and CCR5 molecules and that this initial interaction of the envelope with α4β7 promotes efficient virus capture and facilitates efficient HIV infection of T cells [74]. Uniquely, retroviruses, including HIV, have the ability to convert their genomic RNA into cDNA following entry into a target cell, a reaction catalyzed by the reverse transcriptase (RT) enzyme of the virus [75]. The viral RT catalyzes the conversion of the single-strand HIV genomic RNA into complementary cDNA (Figure 1c). The RNA template strand is then degraded by the RNase H enzyme; the DNA-dependent DNA polymerase activity of RT then converts the single-stranded cDNA into double-stranded DNA, referred to as the provirus. The heterodimeric RT enzyme consists of two subunits: a larger 66 KDa and a smaller 51 KDa subunit. Reserve transcription occurs within the RTC initiated by cellular tRNA, which, after binding to the primer binding site (pbs), leads to the generation of an RNA–DNA hybrid molecule. The U5 region within the 5′ LTR of the viral genome harbors the pbs. The RNase H enzyme, which is part of the RT holoenzyme, then degrades the RNA strand within the RNA–DNA hybrid, creating the DNA minus-strand strong stop [75]. In a process referred to as first-strand transfer, the DNA minus-strand strong stop then shifts to the 3′ ends from the 5′ ends of the RNA to prime the synthesis of the minus strand of the viral cDNA. Fragments of RNA resulting from minus-strand synthesis then bind to purine-rich sequences called polypurine tract (PPT) to initiate the synthesis of the viral plus-strand cDNA, resulting in double-stranded proviral cDNA. Recent studies have, however, demonstrated that there is a dynamic interplay between viral uncoating, reverse transcription and nuclear import [76,77,78]. For instance, a study involving single HIV-1 infection dynamics revealed that the CA protein enables the core docking of the pre-integration complex (PIC) at the nuclear envelope, suggesting that complete uncoating does not occur immediately following the entry of the viral core into the cytoplasm [76]. Similarly, other reports indicated that HIV reverse transcription is intimately linked to CA disassembly, whereby reverse transcription mechanically initiates CA disassembly [77,78].

### 3.2. Integration

Following the process of reverse transcription, HIV cDNA enters the nucleus as PIC with the help of Vpr [67,75]. Nuclear entry of the PIC is mediated by the Vpr through interactions with the host cell nuclear import machinery. The enzyme integrase (IN) within the PIC subsequently catalyzes the integration reaction of HIV cDNA into the chromosome of the host cell. Like all retroviruses and lentiviruses, HIV-1 requires the integration of its cDNA into the host cell DNA. Therefore, the host cell’s metabolic state influences the activity of the integrated provirus [79]. Integration of the HIV cDNA into the host chromosome is a very important step for retrovirus multiplication. For HIV-1, a 32 KDa viral integrase (IN) enzyme catalyzes the integration of viral cDNA into the host chromosomal DNA [67]. In a reaction referred to as 3′ end processing, viral cDNA integration begins with the clipping of several nucleotides from the 3′ end of both strands of the HIV cDNA, generating a cDNA with double strands with 3′-recessed ends. Similarly, IN also cleaves cellular genomic DNA at the sites of integration, after which viral cDNA is ligated to the cleaved cellular DNA ends in a process referred to as strand transfer. The resultant integrated viral cDNA is referred to as the provirus, which essentially behaves like a host gene. The nucleotide gaps between the newly integrated provirus and the host chromosome are repaired by the cellular enzymes to complete the integration process [75]. The HIV provirus now becomes the template for viral mRNA species synthesis, which codes for a full complement of structural, accessory and regulatory proteins of the virus required for replication and virulence [26,80].

### 3.3. Transcription

The HIV-1 LTR contains multiple *cis*-regulatory elements and serves as the site of HIV-1 transcriptional initiation and regulation. The HIV LTR consists of three distinct regions, namely the unique 3′ end (U3), the repeated (R) sequence and the unique 5′ (U5) domains. The promoter region is present within the U3 elements. The promoter mediates the binding of RNA polymerase II (RNAP II) and other crucial components of the transcription machinery. The TATA-box is located at the −28 nucleotide position upstream of the +1 transcription start site. Other important transcription factors, including Sp1 (3 sites) and NF-ĸB (2 sites), are located at the 5′ end of the TATA box [26,80,81]. The binding of a highly conserved 38 KDa TATA box-binding protein (TBP) to the TATA box initiates HIV-1 transcription from the LTR promoter. Additional transcription factors are recruited as a result of the TBP binding to form the pre-initiation complex known as the TBP-associated factors (TAFs). The resultant complex formed is made up of multiple proteins comprising TBP and TAF, referred to as TFIID, which, along with three SP1 binding sites, constitutes the minimal transcription complex that can induce basal HIV LTR promoter transcription. However, for efficient HIV LTR promoter-mediated transcription, it requires the TFIID interaction with upstream enhancer binding factors such as NF-ĸB, AP-1 or NFAT [75,82,83,84,85,86,87,88,89,90]. Transcription factor II H (TFIIH) exhibits kinase activity (CDK7) required for promoter clearance by phosphorylating the C-terminal domain (CTD) of RNAP II, whose recruitment to the HIV LTR promoter is reported to be the major determinant in HIV transcription, especially HIV-1 transcription initiation [80,91,92].

Without the viral Tat protein in the system, transcription of the HIV provirus is not efficient, and transcribing RNAP II that initiates HIV-1 gene expression disengages after a few nucleotides following transcription initiation. However, complete synthesis of HIV-1 mRNA increases when viral Tat is present due to increased efficiency in transcription elongation mediated by Tat, resulting in the synthesis of full-length viral mRNA transcripts [49,50,51]. Tat functions by unusually binding to an RNA element known as the transcription response region (TAR), formed by the first 59 nucleotides. TAR, a stem-loop structure, is present at the 5′ end of all nascent viral mRNA transcripts [80]. When viral Tat binds to TAR, Tat brings P-TEFb, a critical cellular factor that plays a vital role during the transcriptional elongation of cellular genes. P-TEFb predominantly comprises cyclin T1 and the kinase subunit, the cyclin-dependent kinase 9 (CDK9). This kinase component of P-TEFb, CDK9, catalyzes several events, including hyperphosphorylation of the CTD of the largest RNAP II subunit, which makes RNAP II more processive. The processive RNAP II subsequently leads to the generation of complete HIV genomic transcripts required to form new HIV progenies [75].

Transcription from the HIV-1 LTR promoter results in the generation of three categories of viral mRNA transcripts: small, multiply spliced mRNAs (~2 Kb) known to code for regulatory proteins Tat, Nef and Rev of HIV-1; singly spliced mRNAs (~5 Kb) known to code for Env, Vif, Vpu and Vpr proteins; and unspliced RNA (~9 Kb), which acts as mRNA to encode Gag and Gag-Pol polyprotein precursors, in addition to serving as full-length genomic mRNA (gRNA) for packaging into new HIV-1 virion [75,80]. In eukaryotes, mRNAs are spliced in the nucleus before their export for translation in the cytoplasm. Export of large singly spliced or unspliced HIV-1 RNA to the cytoplasm for translation is not usually efficient. To improve export efficiency, HIV-1 possesses a protein called Rev, which binds specifically to the Rev response element (RRE), a *cis*-acting RNA element that mediates the export of singly spliced and unspliced HIV-1 RNA out of the nucleus [75]. The Rev response element spans about 250 nucleotides located within the env gene and folds into a series of smaller stem-loop structures within one central bubble [75]. The RRE is present in all singly spliced as well as unspliced viral RNA transcripts to promote their export into the cytoplasm. Through cooperative protein–RNA and protein–protein interactions, multimers of Rev molecules bind to the RRE within partially spliced or unspliced RNAs to enhance their nuclear export to the cytoplasm.

### 3.4. Translation

Three of the HIV-1 ORF codes for Gag, Pol and Env polyprotein precursors, which are then proteolytically cleaved to form functional individual viral proteins. Unspliced HIV mRNA encodes the Gag (Pr-55 Gag) and Gag-Pol (Pr-160Gag-Pol) precursor polyproteins. Gag Pr-55Gag polyprotein is proteolytically cleaved into p17 or MA, p24 or CA, p7 or NC, and p6 structural viral proteins [67,75] (Figure 1). However, Pr-160Gag-Pol fusion precursor polypeptide is similarly processed to form p10 or PR, p66/51 or RT, and p33 or IN, all of which are the gene products of the pol gene [67,75]. Pr-55Gag and Pr-160Gag-Pol polyproteins are both recognized and processed to form functional viral proteins by the enzyme PR. The gp120 and the gp41 are proteolytic products encoded by the *env* gene. Other proteins of HIV, including virus accessory and regulatory proteins, are translational products of singly and multiply spliced viral mRNAs.

### 3.5. New Viral Progeny

An initial step in the formation of the nucleoprotein complex during virion assembly begins with the recognition and NC binding to the packaging signal, denoted as ψ, located near the gag gene initiation codon [67]. The packaging signal is necessary for the generation of the proper core viral nucleoprotein complex consisting of full genomic RNA, a necessity to form new HIV particles. Given that the packaging signal is removed during splicing, only unspliced complete genomic mRNA (gRNA) is packaged, resulting in new HIV particles. Another component of the nucleoprotein complex is the CA, which assembles in a tabular form and stabilizes the nucleoprotein complex formation [67]. The nucleoprotein core complex consisting of virus gRNA, NC and CA proteins then migrates to the cellular plasma membrane, where it assembles with the Env coat through interactions with N-terminus myristylated MA molecules within the nucleoprotein core. Myrstilation confers hydrophobicity to the nucleoprotein core and promotes its interaction with the lipid bilayer of the cellular cell membrane [67]. The final step in the virion assembly process involves the budding and egress of the new virion particles through the cellular plasma membrane. During budding, the virion acquires a portion of the cell membrane containing viral gp120 and gp41 required for the subsequent infection of the target cell. Gag molecules within new HIV-1 virions then undergo maturation to become fully infectious HIV particles.

## 4. Overview of the Immune Response to HIV Infection and Th1 and Th2 Hypothesis

Upon infection, HIV is carried into the lymph nodes and other lymphoid organs, where enhanced virus replication occurs, leading to systemic infection [93]. During the acute phase of HIV infection, which occurs within six weeks following infection, there is an occurrence of mononucleosis-like syndromes: rash, sores in mouth, fever, pharyngitis, malaise, myalgia, headache, nausea and vomiting, lethargy, ulcers on the genitals, lymphadenopathy, enlarged liver, weight loss, night sweats, diarrhea, and other neurological symptoms, with a substantial increase in viral load in the blood [94]. The increased viremia during the acute phase is concomitant with the decline in CD4+ T-cells attributed to virus-mediated cytotoxicity or cytotoxic T-cells (CTL)-mediated killing of cells infected by the virus [94,95]. The viremia peak normally resolves as a result of HIV-1-specific immune responses; however, this HIV-specific immune response is incapable of suppressing HIV-1 replication [94,95].

The acute HIV-1 infection leads to a chronic asymptomatic phase known as “clinical latency”, which can last for years [95]. The hallmark of clinical latency is the gradual depletion of peripheral blood CD4+ T-cells, which occurs as a result of continued virus replication [96]. Pantaleo et al. reported that though viremia may be low or undetectable in peripheral circulation, virus replication is enhanced in lymphoid organs due to several mechanisms, including viral accumulation, cellular activation and rapid viral turnover, among others [93,97,98]. T-cell depletion results from direct virus-mediated cytotoxicity and infection-induced CTL-mediated killing of HIV-infected cells [93,97,98]. It has also been observed that HIV-1 infection induces autoimmunity during the entire course of infection, resulting in hyper-activation of cellular immunity, which then causes the nonspecific killing of immune cells [95].

The prolonged period of clinical latency marked by a progressive decline in host immunity causes the inability of the host to respond to other invading pathogenic microorganisms and is known as acquired immunodeficiency syndrome (AIDS) [99]. The acquired inability of the host to induce an immune response during the AIDS phase results in the onset of a range of opportunistic infections that become associated with HIV-1 infection referred to as AIDS-defining illnesses [94,95,99].

Helper CD4+ T cells, which can be broadly categorized into T-helper 1 (Th1) and T-helper 2 (Th2) subsets, are responsible for the activation of HIV-specific adaptive immune responses. The Th1 and Th2 hypothesis was initially proposed in 1986, suggesting that the T-helper cells from mice expressed different cytokine profiles [100]. Subsequently, in 1989, Mosmann et al. reported that this hypothesis was adapted to human immunity, whereby Th1 and Th2 cells were suggested to mediate differential immune responses [101]. In this case, Th1 cells mediate the type-1 pathway, also known as cellular immunity, which fights intracellular pathogens such as viruses, prevents cancer cell formation and promotes delayed-type hypersensitivity. On the other hand, Th2 cells direct the type-2 pathway, also known as antibody-mediated (humoral) immunity, which fights extracellular pathogens such as bacterial infections.

### 4.1. Mechanism of Th1 and Th2 Classification and Differentiation

The classification of T-helper cells into Th1 and Th2 is based on the cytokine profiles produced by these T-cell subsets. Th1 secret Interleukin 2 (IL-2), Interferon-gamma (IFN-γ) and Tumor necrosis factor-beta (TNF-β). Meanwhile, Th2 cells secret Interleukin 4 (IL-4), Interleukin 5 (IL-5), Interleukin 6 (IL-6), Interleukin 10 (IL-10) and Interleukin 13 (IL-13) [102]. In 1994, Fishman and Perelson observed that Th1 and Th2 polarization was a result of the Th1/Th2 cross-regulation [103]. In this case, IFN-γ produced by Th1 cells prohibits Th2 cell proliferation. On the other hand, IL-10 produced by Th2 cells prohibits Th1 cells' cytokine production. Therefore, based on this model, immune responses are dominated by either Th1 or Th2 cells but not both. The arm of the immune response that dominates is determined by the relative activation efficiencies of Th1 and Th2 cells. However, it is important to note that any disturbances to the system by environmental factors allow a switch between the two arms of the Th1 to Th2 cells. In light of this observation, several disease conditions are known to cause a shift from Th1 to Th2 or vice versa. Examples include Atopic Dermatitis, marked by Th1 to Th2 imbalance in mesenchymal stem cells in the early phase of the disease [104], Peritonitis, exposure to endotoxins, respiratory syncytial virus (RSV) infection [105], among others.

Following the encounter with an antigen, CD4+ T cells can differentiate into the Th1 or Th2 cell subsets. Differentiation into Th1 or Th2 is mediated by linage-determining transcription factors, whereby Th1 differentiation is mediated by the T-box transcription factor (T-bet), while Th2 differentiation is mediated by GATA-binding protein 3 (GATA-3) transcription factors [106,107]. Whereas T-bet is expressed only in Th1 cells, GATA-3 is expressed in both Th1 and Th2 cells, and both transcription factors share target genes, particularly in Th1 cells. Therefore, in Th1 cells, the choice between Th1 and Th2 lineages occurs as a result of the opposing effects of T-bet at the shared target genes. Previous murine studies demonstrated that Th1 differentiation occurred due to acetylation of histone H3 lysine 9 (H3K9ac) of the *IFNG* gene locus, which is mediated by T-bet. In contrast, Th2 cell differentiation was accompanied by hyperacetylation of the *IL-4*, *IL-5* and *IL-13* gene locus mediated by GATA-3 [108]. Other studies indicated that T-bet directly represses *IL-4* while GATA-3 directly represses *IFNG*. Most recently, Hertweck et al. demonstrated that in Th1 cells, T-bet directly interacts with GATA-3, with the result that GATA-3 is sequestered from its canonical binding sites to drive the Th1 gene expression program while silencing the Th2 gene expression mediated by GATA-3 [107]. These results indicate that the presence of T-bet and GATA-3 regulates Th1 and Th2 cell differentiation, respectively.

### 4.2. Mechanism of Th1/Th2 Shift in HIV Infection and Preferential Virus Infection of Th2 Cells

**Figure 2:**
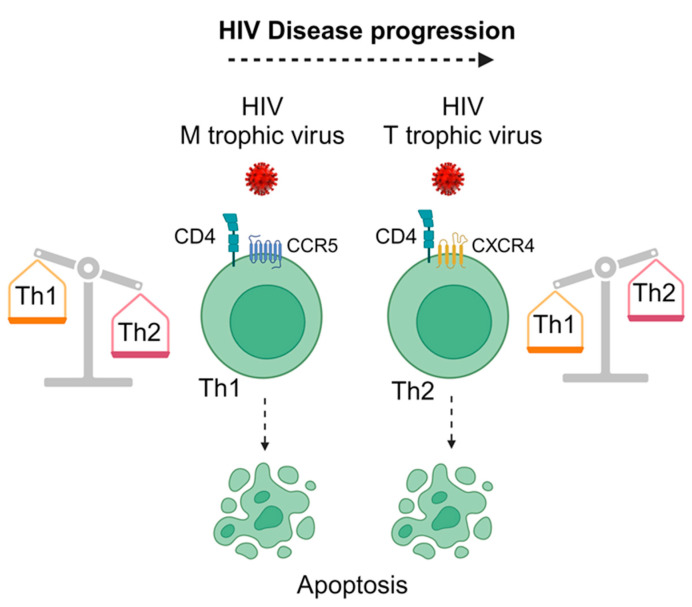
Schematic diagram illustrating the differential infection and depletion of the Th1 and Th2 T-cell subsets by HIV-1 during the course of HIV disease. During the early asymptomatic stage of HIV infection, the M-trophic CCR5-utilizing viral strains predominate infection, whereby the Th1 cells are massively infected and depleted due to apoptosis. On the other hand, the T-trophic CXCR4-utilizing HIV strains, which transmit ineffectively and are rarely found during the early asymptomatic phase of HIV disease, become predominant during the late symptomatic stage of HIV disease, whereby preferential infection of Th2 cells occurs, resulting in their massive depletion. This figure was prepared using BioRender software (https://app.biorender.com/ (accessed on 23 November 2023)).

The Th1 to Th2 shift has been reported during the HIV infection [109]. However, considering the foregoing paragraph, this phenomenon is not unique to HIV but also occurs in a number of other disease conditions. Based on the findings that (1) HIV-1 disease progression is characterized by loss of production of IL-2 and TNF-α concomitant with increased IL-4 and IL-10 production and (2) HIV-exposed seronegative individuals (HESI) exhibited a strong Th1 response, Clerici and Shearer proposed that the Th1 to Th2 switch in HIV infection contributes to the dysregulation of the immune system, such that resistance to HIV infection or HIV disease progression is determined using the Th1 dominance [109]. Perhaps the intriguing question to ask is what mechanism mediates this T-helper cell polarization from Th1 to Th2 cells during HIV infection? Although Becker reported that the Th1 to Th2 shift during HIV infection was caused by an allergic reaction in HIV-infected individuals as a result of exposure to allergens in the environment [110], there is a lot of inadequacy in this observation. In this review, we explored this question and came up with a novel observation and a new mechanism to explain it. We observed that the differential expression of HIV coreceptors by Th1 and Th2 cells confer preferential infection of T-cell subsets by HIV-1 during the HIV disease course. Firstly, to better explain this and understand the mechanism, we need to understand HIV-1 tropism and coreceptor usage during HIV-1 infection. HIV-1 tropism is defined as the capacity of the HIV to infect alternative CD4+ cell subsets, which is determined using the HIV coreceptor usage. For instance, M-trophic viruses primarily infect macrophages and CD4+ T cells using the CCR5 HIV coreceptor. On the other hand, T-trophic viruses can infect CD4+ T cells and T-cell lines using the CXCR4 HIV coreceptor. Viruses with dual-tropism exhibit both M-trophic and T-trophic characteristics using the CCR5 and/or CXCR4 coreceptors [111]. Changes in the composition of viruses with different tropisms have been observed during HIV infection. For instance, during the early asymptomatic stage of HIV infection, CCR5-utilizing strains have been shown to predominate [112,113,114,115]. On the other hand, the CXCR4-utilizing HIV strains, which transmit ineffectively and are rarely found during the early asymptomatic phase of HIV disease, instead become predominant during the late symptomatic stage of HIV disease [112,114,116,117]. Because CXCR4-utilizing HIV strains are correlated with the symptomatic stage of HIV infection, characterized by CD4+ T cell decline and onset of clinical symptoms, it is believed that they are more pathogenic than the CCR5-utilizing HIV strains, but this is not necessarily true. Firstly, the differences in chemokine receptor expression apparently explain the shift from Th1 to Th2 immune response during HIV disease progression. In this case, different chemokine receptors have been found to be differentially expressed on Th1 and Th2 cell subsets. For instance, CCR5 coreceptors were expressed preferentially by the Th1 cells, while CXCR4 was expressed preferentially by the Th2 cells [118,119]. This observed differential expression of HIV coreceptors by Th1 and Th2 cell subsets would imply that there is preferential infection of the Th1 and Th2 cells by HIV-1 during the HIV disease course. Considering the fact that CCR5-utilizing HIV-1 strains are predominant during the early asymptomatic stage of the HIV disease, this would conceivably imply that CCR5-utilizing viruses preferentially infect Th1 cells first, which cells would massively die as a result of the cytotoxic effector mechanisms of the immune cells in addition to the virus cytopathic effects (Figure 2). During the late symptomatic stage of HIV-1 disease, when CXCR4-utilizing HIV strains are most predominant, then the infection dynamics shift whereby Th2 cells are mostly infected by CXCR4-utilizing viruses [120]. Accordingly, this would appear as if HIV-1 infection causes a Th1 to Th2 shift, but then this apparent shift is brought about by the differential HIV coreceptor expression on the Th1 and Th2 cells, which also results in the preferential HIV-1 infection of these two T-helper cell subsets. Our analysis is consistent with the observation of Romagnani et al., whose alternative viewpoint to the Th1 to Th2 shift hypothesis established that there was indeed no physiological in vivo induction of the Th2 state in HIV infection [121].

### 4.3. The Th1/Th2 Shift in the Context of HIV and Co-Infections

Infection by HIV produces a progressive weakening of the immune system of the host because of the massive depletion of the CD4+ T cells involved in host defense. The progressive decline in host immunity is associated with the occurrence of different co-infections, which have been reported in HIV patients. These co-infections may exhibit different effects on the Th1/Th2 balance. One of the most common co-infections in HIV disease is *M. tuberculosis* infection, which occurs as an opportunistic infection. Being an intracellular pathogen, *M. tuberculosis* infection is reported to exhibit a higher Th1/Th2 ratio [122]. Interestingly, chronic HIV infection causes a shift from Th1 to Th2, resulting in a decrease in the Th1/Th2 ratio due to the cytokine imbalance. This Th1 to Th2 shift caused by HIV infection impedes the activation of cytotoxic T cells (CTLs), which are critical for the clearance of intracellular HIV and *M. tuberculosis*. Furthermore, HIV infection results in the depletion of CD4+ T cells, which, when activated, release IL-2 required for both CD4+ and CD8+ T cell proliferation. Low CD4+ T cell counts increase the risk of opportunistic infections, including *M. tuberculosis.* Ultimately, the Th1 to Th2 shift and the concomitant decline in CD4+ T cells result in the inability of the immune system to clear *M. tuberculosis* infection [122]. On the contrary, during HIV and *M. tuberculosis* co-infection, *M. tuberculosis* is reported to secrete the EspR protein, a transcription regulator that localizes into the cell nucleus where it binds to the promoter region of the IL-2 gene, resulting in high expression of the IL-2 protein. The increased IL-2 expression causes a Th1 to Th2 shift, which not only compromises the clearance of intracellular *M. tuberculosis* but also favors HIV pathogenesis [123]. In a study that assessed the cytokine serum levels in patients infected by HIV with or without *T. cruzi* co-infection, results indicated that there was a Th1 to Th2 shift apparently due to HIV infection. However, the Th1 to Th2 shift was associated with the presence of parasitemia in the case of HIV and *T. cruzi* co-infection, suggesting that the Th2 shift favored parasite multiplication [124]. In another study that investigated the effects of HIV and *S. mansoni* co-infection on HIV-specific Th1 immune response, results indicated an enhanced Th1 response in HIV and *S mansoni* co-infected individuals compared to HIV-only infected individuals, suggesting that *S. mansoni* infection enhanced Th1 response. Proinflammatory cytokines such as TNF-α and IFN-γ associated with Th1 response are likely to exacerbate HIV pathogenesis, such that *S. mansoni* co-infection likely favors HIV disease progression by enhancing immune activation [125,126].

### 4.4. Implications of Early HIV Treatment Initiation and Care

Since Zidovudine (AZT) was introduced in 1987, HAART evolved from monotherapy to dual therapy and now comprises a combination of three drugs from at least two different classes of antiretroviral agents [127]. The optimal time for HIV patients to initiate HAART has also evolved over time. Initially, the guidelines on when to initiate HAART and access HIV care differed from country to country. Overall, treatment was predominantly reserved for symptomatic patients [128]. In 2010, the World Health Organization (WHO) recommended that HAART should be initiated when the CD4+ T cell count is less than 350 cells/mm^3^. Then, in 2013, informed by the HPTN 052 study, the WHO again recommended that HAART should be initiated earlier when the CD4+ T cell count is less than 500 cells/mm^3^ [129]. Subsequently, in 2015, following evidence presented by the START and TEMPRANO studies, which reported better treatment outcomes following early HAART initiation, the WHO recommended that all HIV patients should initiate HAART following diagnosis, regardless of the status of the CD4+ T cell counts. It should be noted that during the acute phase of HIV infection, there is an overall increase in the T cell population triggered by HIV replication in blood and lymph nodes, which is able to temporarily control viremia in the short term. However, this increase in the T cell population is short-lived as increased virus replication results in massive depletion and decline initially in Th1 cells, followed by the Th2 cell subsets, as discussed in the foregoing section. Therefore, the initial policy of the WHO, which allowed for the initiation of HAART when the T cell count was below 500 cells/mm^3^, implied that HAART would be initiated at a time when the vast majority of the Th1 cells had already been depleted. Yet, HIV being an obligate intracellular pathogen, it is also this Th1 response (cell-mediated immunity) that is responsible for the control of intracellular HIV infection. This implies that (1) there would be poor immune control of the HIV infection and (2) there would be poor restoration of the Th1 cell subsets following the late initiation of HAART.

Infection of mucosal tissues by HIV-1 triggers a series of pathologies that subsequently affect both the integrity and function of the epithelial barrier and the microbiota composition. For instance, in the gut and lymphoid tissues, this involves the destruction of epithelia, bacterial translocation and depletion of follicular helper CD4+ T cells, loss of the architecture and function of the germinal centers, immune cellular metabolic reprogramming and inflammation [130,131,132,133,134]. Furthermore, HIV-1-induced disruption of the germinal centers and the subsequent impairment of the antibody response also favor the translocation of invading microbial products, chronic immune activation, and an imbalanced ineffective antibody response [135,136,137]. Therefore, early initiation of HAART during primary infection is shown to prevent HIV-1-induced mucosal damage and immune dysregulation [138,139]. Furthermore, early HAART initiation enables the preservation of functional gut follicular helper CD4+ T cells and Env-specific memory B cells, which facilitates the development of functionally relevant HIV-1 antibodies [140,141,142]. Most recently, Planchais et al. investigated the impact of HAART initiation timing on the intestinal B-cell antibody repertoire from colon tissues of individuals treated either during the acute (eART) or chronic (lART) phases of infection [143]. They demonstrated that HIV-1 HAART treatment timing shapes the systemic and gut mucosal memory B-cell repertoire. In this case, delayed HAART initiation is associated with an increase in poly- and commensals-reactive memory B cells in the gut mucosa, which subsequently spreads to the periphery and circulate in the blood where there are high levels of bacterial translocation, inflammation and commensal-binding immunoglobulin markers. Early HAART initiation attenuates HIV-1-associated pathological events in the gut mucosa, especially the translocation of commensal bacteria, which triggers antimicrobial antibody and memory B-cell responses, thereby preventing the development of abnormal and potentially deleterious humoral responses to commensal bacteria at both mucosal and systemic levels.

Therefore, our observation, and indeed the mechanism to explain it, supports the current WHO recommendation to initiate HAART upon HIV diagnosis. However, the key question here is when does HIV diagnosis take place? In developing countries, HIV diagnosis has always been linked with the development of AIDS symptoms, whereby individuals were tested following the presentation of AIDS symptoms or when presenting with opportunistic infections associated with HIV/AIDS, such as tuberculosis. However, this practice has since changed following the initial introduction of the 90–90–90 policy of the United Nations Program on HIV/AIDS (UNAIDS). This policy aims for 90 percent of all people living with HIV to know their HIV status, 90 percent of HIV-positive individuals to be initiated on HAART, and 90 percent of those on HAART to be virally suppressed. Early diagnosis implies that there should be availability and improved access to HIV counseling and testing, concomitant with referral to HIV treatment and care services to enable the early initiation of HAART. Early initiation of HAART allows for the restoration of the function of both the Th1 and Th2 T cell subsets before these cells are completely depleted, thereby improving the overall T cell population and function. This would significantly reduce comorbidity due to infection with opportunistic illnesses associated with the late symptomatic stage of HIV infection.

## 5. Conclusions

The HIV/AIDS pandemic remains a major challenge in the world. However, in the last three decades, concerted efforts have led to the development of prevention strategies for combating HIV/AIDS. In this review, we update the understanding of HIV molecular biology, including the viral life cycle and the Th1 to Th2 shift that occurs during HIV infection. We also discuss the preferential infection of Th2 cells by HIV during the late symptomatic stage of the HIV disease and its implications for the early initiation of HIV treatment and care. Immunity is designed to respond to both intracellular pathogens mediated by cellular immunity induced by Th1 responses and extracellular pathogens mediated by humoral immunity induced by Th2 cells. The two T-helper cell subsets, Th1 and Th2, secret different cytokine profiles, which cross-regulate each other. In addition to cross-regulation, different disease conditions are reported to shift the balance between Th1 and Th2 responses. In the case of HIV infection, a shift from Th1 to Th2 responses have been observed. However, this Th1 to Th2 shift is not caused by a mechanistic change in T-helper cell differentiation but rather by the differential expression of the CCR5 and CXCR4 chemokine receptors, which also function as HIV coreceptors. This differential expression allows for the preferential infection of the Th1 and Th2 subsets at different stages of the HIV disease. In this case, Th1 cells are preferentially infected and depleted during the early asymptomatic stage of HIV infection. Th2 cells, on the other hand, are preferentially infected during the late symptomatic stage of the HIV disease. It is imperative to note here that the early preferential infection and depletion of Th1 cells, which mediate cellular immunity responsible for fighting intracellular pathogens such as viruses, including HIV, could be responsible for exacerbating HIV disease progression. Our analysis has implications for when HAART should be initiated. Consistent with the current recommendation of the WHO, we recommend that HAART should be initiated early to prevent the depletion of Th1 cells, which preferentially occurs very early following HIV infection. This would improve overall T cell function and prevent comorbidities caused by opportunistic infections associated with HIV disease.

## Figures and Tables

**Table 1:** Characteristic differences between HIV-1 and HIV-2.

Characteristics	HIV-1	HIV-2	References
Origin	Chimpanzee	Sooty mangabey	[10,11,12]
Strain	Predominant	Rare	[13,14]
Pathogenicity and infectivity	High	Low	[4,15]
Viral replicative fitness	100-fold more fit	Less fit	
Genetic diversity	Diverse	Low	[16,17,18]
Transmission	High	Low	[19,20,21]
MTCT	High	Rare	[20,21]
Prevalence	Worldwide	West Africa	
Blood plasma viral load	High	Low	[2]
Mortality rate	High (87%)	Average (52%)	[22]
CD4 count at time of AIDS	Below 100 cells/µL	Above 100 cells/µL	[22]

MTCT: Mother-to-child transfer.

**Table 2:** Genes of HIV-1, their protein products and functions.

Type	Gene	HIV Protein	Main Function	Refs.
Structural	*env*	gp120 gp41	Binds to HIV receptor, CD4 molecule and CCR5 and CXCR4 co-receptors of host Promotes virion fusion to host cell	[30] [31]
	*gag*	P24 P17 P7 p6	Capsid protein Matrix protein Capsid protein Capsid protein	[32] [33] [33]
	*pol*	RT IN PR	Converts viral RNA into dsDNA Integration of dsDNA into host Cleavage of gag and pol precursors	[34] [35]
Regulatory	*tat*	Tat	Promotes proviral DNA transcription	[36,37,38]
	*rev*	Rev	Exports unspliced viral RNA to cytoplasm	
	*nef*	Nef	Downregulation of CD4, MHC-1 and other receptors	[39]
Accessory	*vpr*	Vpr	Regulation of PIC nuclear import	[40]
	*vif*	Vif	Promotes virion infectivity	
	*vpu*	vpu	Intracellular CD4 degradation	[41]

PIC: Pre-integration complex; MHC: major histocompatibility complex.

## Data Availability

All the data is included in the manuscript.
